# Are habitually barefoot children compelled to wear ill-fitting school shoes? A cross-sectional study

**DOI:** 10.1186/s12887-022-03263-9

**Published:** 2022-04-08

**Authors:** Marise Carina Breet, Ranel Venter

**Affiliations:** Movement Laboratory, Department of Sport Science, Faculty of Medicine and Health Sciences, Stellenbosch University, Stellenbosch, South Africa

**Keywords:** Shoe fit, Foot development, Children, Barefoot, School shoes

## Abstract

**Background:**

Research shows that ill-fitting shoes can negatively impact the development of the pediatric foot, in a very direct manner. The primary aim of the study was to determine if the dimensions of available prescribed school shoes fit the foot dimensions of habitually barefoot South African children and adolescents.

**Methods:**

A cross-sectional observational study was conducted where static standing foot measurements of children and adolescents from urban and rural schools were obtained with a mobile caliper. The maximum heel-toe-length and foot width with an added 10 mm toe- and width fit allowance to each participant, were compared to the corresponding school shoe length and shoe width available in retail. A mixed model ANOVA was used to compare foot dimensions between gender, age and side.

**Results:**

Six hundred and ninety-eight school children (*N* = 698) (431 girls; 267 boys; average age 10.86 years, SD = 2.55) were participants. A total of seventy-seven (*N* = 77) black coloured prescribed school shoes currently available in retail ranging from different styles and brands were measured. Results show that, comparing the shoe length and maximum heel-toe-length of participants, as well as taking 10 mm toe allowance into account, fifty-nine percent (59%) of children wore shoes that were not the correct length. With regards to the shoe width and the added 10 mm of width fit allowance, ninety-eight percent (98%) of the shoes worn by participants were too narrow for their feet.

**Conclusions:**

Results confirmed that school shoes currently available in retail, are not suited for the habitually barefoot population studied. It is recommended that the shoe manufacturing industry should consider the shoe width of school shoes for children and adolescents in habitually barefoot populations to avoid the long-term negative effect of ill-fitting shoes on the pediatric foot.

## Background

Foot morphology has been studied in various populations over the years. In recent years the function and development of the foot have sparked renewed interest [1]. Areas on which studies have focused over the past ten years include foot movement patterns [2–6], the advantages and disadvantages of barefoot locomotion compared to shod walking [6–11], shoe development, shoe characteristics, shoe fitting assessment methods [12–17], and inter-continental differences in foot morphology and foot function [1, 7, 17–19]. A few recent studies have focused on the pediatric foot, and specific footwear habits and foot development [2, 7, 14, 20]. It has been emphasized that the child’s developing foot may have the same features as an adult foot but should not be treated as such. Ongoing development and specific needs, through different stages of growth, make the pediatric foot vulnerable to internal and external influences [15]. The soft tissues and foot bones are still maturing during these stages, and the full development of these structures are only achieved late in adolescence [21]. Internal factors such as age, gender, and body mass index (BMI) influence the development of the pediatric foot. Rapid changes in foot shape and function throughout the first fourteen years of life, coupled with high variability in static- and dynamic positions, have been reported [3, 5, 20, 22]. During standing and walking increased body height and weight lead to more foot loading and peak pressure in different areas of the foot [3]. A wider midfoot section and flatter foot arch were observed in boys throughout the first nine years of development [3]. During the corresponding developmental stage, higher foot arches were observed in girls [23].

Most notable external factors influencing the development of the pediatric foot are climate, socio-economic status and shoe-wearing habits [18, 19, 22, 24]. For example, growing up barefoot, compared to growing up shod, influences pediatric foot arch morphology, foot pliability, the hallux valgus angle, rearfoot strike patterns and motor performance [6, 7, 25]. Shoes have been identified as an external factor that could significantly influence foot development, as well as gait in children [9, 11].

Forefoot movement patterns are also significantly influenced by shoes [9]. Compared to barefoot walking, increased rearfoot strike patterns and knee and ankle range of motion, as well as longer steps, but reduced foot movements, swing phase leg speed and shock absorption, were reported in children wearing shoes [11]. Although shoes should primarily protect the foot from the external environment, they should still allow the foot to develop and function optimally [12, 15, 16, 22, 23]. The impact of shoes on developing feet is illustrated by the fact that shoes are often used to treat foot deformities and certain musculoskeletal injuries [12]. Therefore, it is important that shoes are developed to fit properly, and not to interfere with the development of the pediatric foot.

Ill-fitting shoes have been associated with foot deformities such as hallux valgus and hammertoes, as well as heel spurs, plantar fasciitis, foot ulcerations and foot arthritis. These foot problems can lead to changes in the biomechanical alignment of the body and impact on load transfer during walking, causing other musculoskeletal conditions and pain, such as knee osteoarthritis and low back pain [8, 12, 13]. It has been recommended that the fitting of shoes, as well as shoe characteristics, should be considered by health professionals when treating patients with musculoskeletal injuries [12]. Large portions of adults and children in Spain, Germany, the United Kingdom and South Africa are wearing ill-fitting shoes [10, 13, 14, 18, 21, 26], with associated pain and foot pathology [16, 27, 28]. Previous research, mostly in adult populations, have shown that 46–81% of participants wore shoes that were too narrow for their feet [16]. Most comprehensive studies have been done on adult feet and adult shoe design [29] and not the pediatric population.

There are several possible reasons for the prevalence of ill-fitting shoes. Recent research has indicated toe- and width allowances are not applied effectively. Often too much or too little space is left for the toes inside the shoe to ensure a correct shoe fit [10]. The recommended toe allowance of 10 mm (millimetres) should be considered in addition to the HTL (heel-toe-length) of the child to guarantee the correct fit for SL (shoe length) [10]. According to Barisch-Fritz et al*.* [10] toe allowance is the same for all shoe sizes and genders. A width allowance of 10 mm should be considered when addressing the fit for the SW (shoe width) [14]. An important factor is that the development of children’s shoes is often not predominantly influenced by orthopaedic and biomechanical considerations, but on fashion trends [10, 15, 21]. Shoes currently available in retail tend to focus on the correct fit in SL but limited guidelines are given regarding the width of the shoe [10]. South Africa’s shoe manufacturers use a shoe design based on the British system, using HTL as the basic measurement. In this system, each increase in HTL will correspond with a standardized increase in foot girth, based on the Mondo Point System [28, 30]. Thompson et al*.* [28] found that sixty-two percent (62%) of adult female participants in a study conducted in South Africa had a forefoot length-to-girth greater than the standardized length-to-girth ratio [10, 15, 21]. Differences in inter-continental foot morphology should be considered when developing shoes for children. This should ensure the correct shoe fit according to the unique foot dimensions of the child, based on previously mentioned external factors [18, 19]. Unfortunately, a lack of available information on inter-continental foot morphology differences contributes to the current problems with child-friendly shoes [18]. Researchers emphasize that the functional aspects of the shoe should cater for unhindered, age-appropriate development of the foot [13–15, 31].

Mauch et al*.*[18] have stressed the importance of obtaining comprehensive information on the differences in foot morphology in children across different continents, including habitually barefoot populations. South African children have an inherent culture of walking barefoot, with a significant majority of children between the ages of six and eighteen years reported to be habitually barefoot [7]. Warmer climates allow children to be barefoot more often, during most parts of the year, which could influence foot development and foot morphology. For example, differences between the foot morphology of South African and German children have drawn attention to on a higher medial longitudinal arch, longer HTL, wider FW, and a difference in foot pliability in the South African study population [32]. There has been limited research on school shoe fit in habitually barefoot children and adolescents [26]. In South Africa, school shoes typically form part of the prescribed school uniform. The aim of this study was to determine if the length and width dimensions of new school shoes available in retail are compatible with the foot dimensions (length and width) of habitually barefoot South African children and adolescents.

## Methods

A cross-sectional observational study was conducted, with foot measurements taken of children and adolescents between the ages six and sixteen, from both urban (*N* = 379) and rural (*N* = 319) schools in a large region of South Africa. After receiving approval from the Department of Education, schools were randomly selected per stratum (representing a combination of regional and school models). These selected schools were contacted via email by the principal investigators.

All children and adolescents from each school, as well as their parents or legal guardians, were informed about the study through a Written Project Information Sheet. Consent forms were handed out two weeks prior to the commencement of testing. Inclusion criteria stipulated a) boys and girls between six and sixteen years of age, b) who attended one of the participating schools in the Western Cape, c) who had submitted completed and signed Informed Consent and Assent forms. Participants were explicitly excluded if they had an acute foot injury at the time of testing or a severe foot deformity, in which case their feet could not be measured accurately. All participants were assessed for height and weight and self-reported their age and gender. Their shoe sizes at the time of data collection were self-reported, as well as documented by the researcher as seen inside the shoe. All measurements were taken once-off during school hours. Ethical approval was obtained from the Research Ethics Committee of the institution (REC-2018–7153) and the Health Research Ethics Committee (Project ID 14,419, Ref number S20/01/008). The study was carried out in accordance with the Helsinki Declaration guidelines.

### Foot measurements

**Fig. 1:**
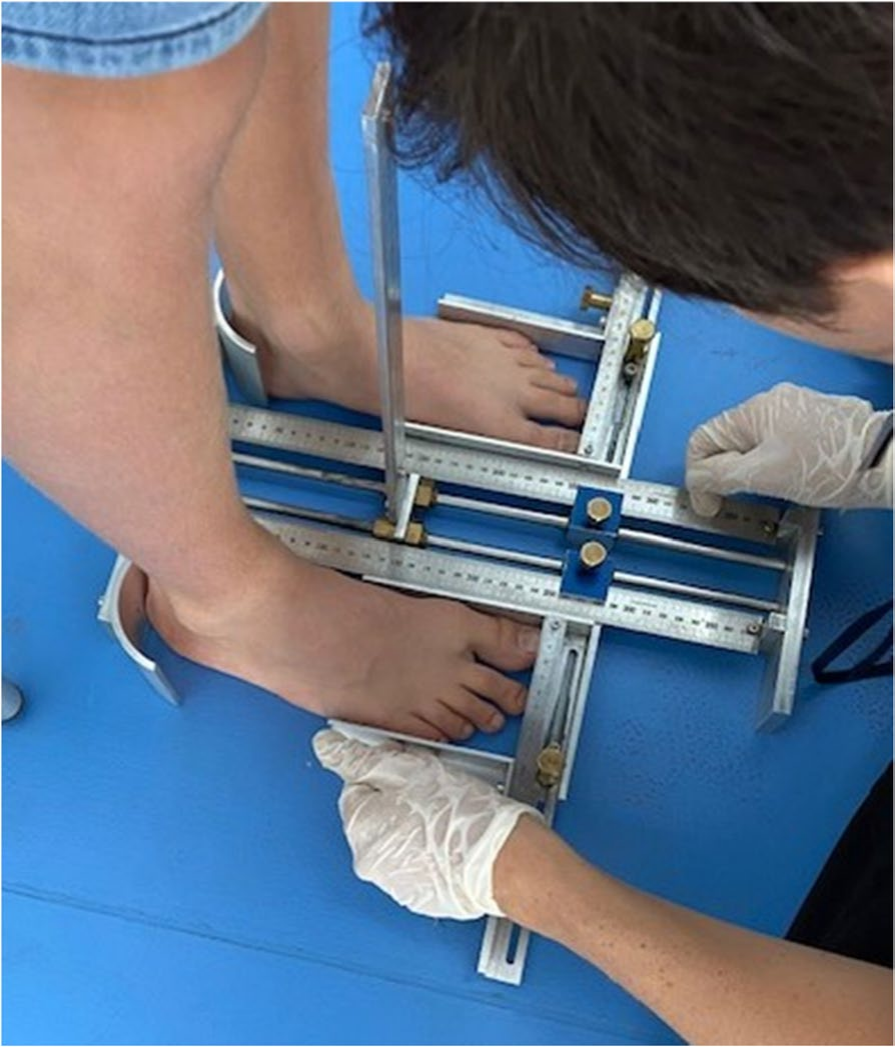
Heel-to-toe-length and foot width measured with a specially constructed caliper

Static foot measurements were done with a specially constructed foot caliper, used in previous research [7, 24, 26]. The caliper consisted of heel cups for proper positioning and horizontal metal sliding indicators for accurate measurement, with a resolution of 1 mm [24] (Fig. 1). The reliability of this static foot measurement was shown to be in the range of “good to excellent” for children and demonstrated high reproducibility [33].

The foot HTL and FW were measured barefoot with the participant standing with the back of the heels touching the heel cups, both knees extended, and weight distributed evenly between both feet and looking straight ahead. In the standing position, the foot being measured is in an elongated position, with fifty percent (50%) of the body weight distributed over each foot. For the purpose of this study the researchers wanted to obtain the largest measurement of HTL and FW to determine the static dimensions which should be accommodated by shoes [12, 22]. Both the right and left feet were measured. Measurements of the longest and widest feet were used for statistical analysis, with a coin flipped to determine the use of the measurements when both feet were of equal length or width [14]. HTL was defined as the distance, in millimetres, between the most posterior aspect of the foot and the most anterior part of the toes [22, 24]. FW was measured, in millimetres, between the most medial part of the first metatarsal head (MTH1) and the most lateral point of the fifth metatarsal head (MTH5) [22]. All measurements were recorded to the nearest 0.01 mm.

### Shoe measurements

**Fig. 2:**
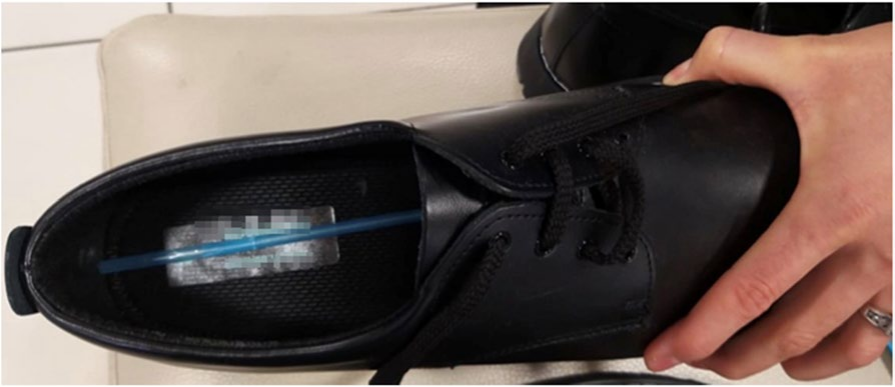
The image shows a plastic straw cut to fit the length of the shoe

Measured sizes of new shoes ranged from United Kingdom (UK) child size number twelve (12) to UK adult size number ten (10) for boys. The girls’ shoes ranged from UK child size number ten (10) to UK adult size number eight (8). Four different brands of shoes, readily available in the retail sector, were measured. No health branded shoes or shoes for special populations were included in these measurements. Measurements of SL and SW were performed on the right shoe of new school sizes. A flexible plastic straw was used to measure the inside length of the shoe according to the guidelines recommend by Barton et al*.* [12] (Fig. 2). One end of the straw was placed in the toe area at the longest part of the shoe, with the other end, touching the heel cup. The straw was then bent at the heel and cut [12].

The straw was measured to the nearest millimetre using a steel ruler. An additional 5 to 20 mm toe allowance to the HTL measurement [12, 14, 15] has previously been suggested for the splaying and elongation during movement of the developing foot [12]. This is to ensure a proper fit of the foot in the length of the shoe. In the current study, the researcher performed standing static HTL measurements, and measured the foot during maximal extension. A toe allowance of 10 mm was added to the HTL to ensure SL fit.

**Fig. 3:**
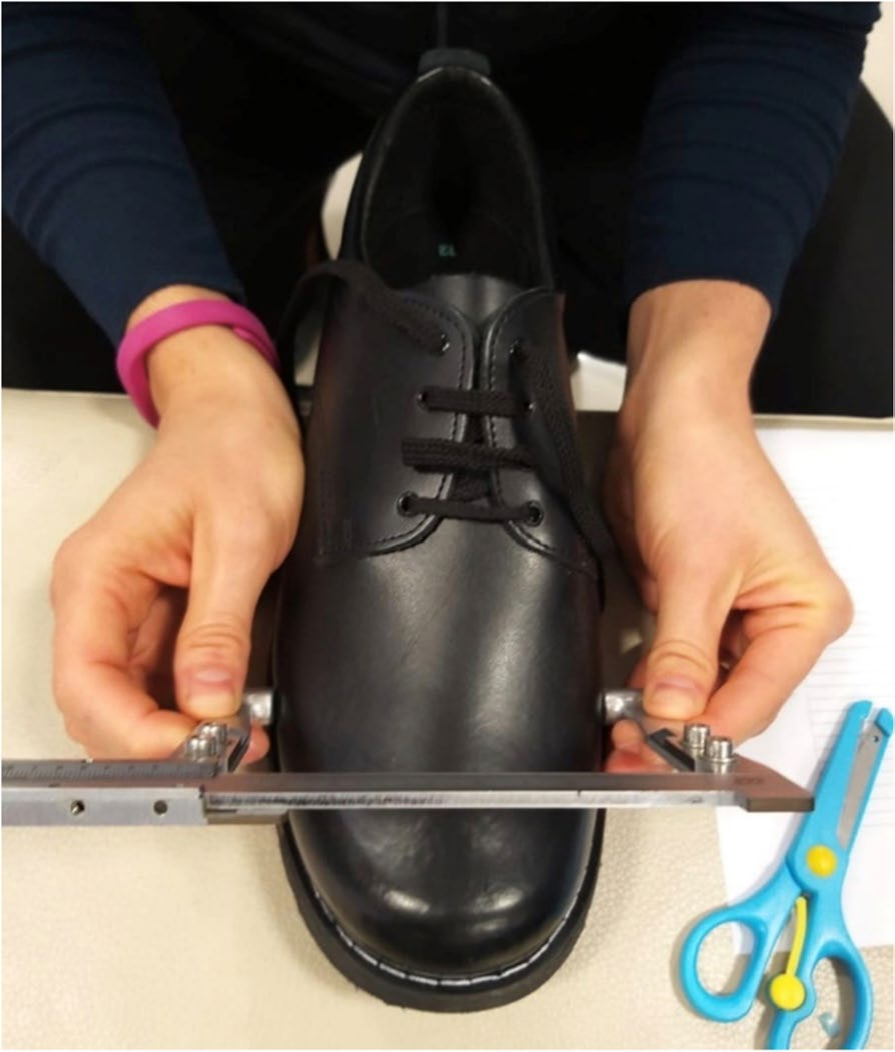
Shoe width measurement with caliper

The width of the shoe was measured using a sliding caliper [34]. The caliper was placed over the upper part of the shoe, and the measurement was taken on the most medial and lateral parts of the shoe. Specific care was taken not to compress the shoe during measurement (Fig. 3). A SW allowance of 10 mm has been suggested to ensure a proper fit [14].

The participant’s shoe size and foot size were available to compare to the range of new school shoe sizes available. The HTL measurement with the added toe allowance of 10 mm of each participant were compared to the ranges of SL of the corresponding new schools in retail of the same size the participant reported wearing the day of testing. The same method was used comparing the FW with the added width allowance of 10 mm with the SW of the corresponding new school size.

Four different brands of new school shoes were measured with sizes ranging from a child’s size ten to an adult’s size ten shoe, including separate measurements of shoes for boys and girls. It became evident that the sizes of the various brands differed, e.g., the length and width of a size three shoe would be different between brands. Ranges for shoe width (SW) and shoe length (SL) were compiled, based on the most narrow and shortest shoe to the widest and longest shoe for each size of the various brands. Each child’s foot measurements and current shoe size were compared to the corresponding new school shoes sizes available in retail. Three groups for shoe fit, based on comparisons between foot length, foot width and the ranges for shoe length and shoe width, were created (Table 1).

**Table 1:** Groups for shoe fit based on comparisons between HTL, SL, FW and SW

**Groups based on comparison between foot length and shoe length**	
Too short	HTL larger than ranges for SL of corresponding shoe size
Within limits	HTL within limits of the ranges for SL of corresponding shoe size
Too long	SL ranges larger than HTL for corresponding shoe size
**Groups based on comparison between foot width and shoe width**	
Too narrow	FW larger than ranges for SW of corresponding shoe size
Within limits	FW within limits of the ranges for SW of corresponding shoe size
Too wide	SW ranges larger than FW of the corresponding shoe size

### Reliability of foot and shoe measurements

Two researchers completed a test–retest reliability assessment for both intra- and inter-tester reliability. The researchers did not have access to their previous measurements. Inter-rater reliability was calculated with an interval of one week. Inter-rater intraclass correlation coefficient (ICC) was 0.99 for HTL Left, 0.98 for HTL Right, 0.99 for FW Left, and 0.93 for FW Right. Inter-rater ICC was 0.99 for SL, and 0.99 for SW.

### Statistical analysis

Statistical analyses were done using Statistica 13.3.0 (TIBCO Software Inc, Palo Alto, CA, USA). Mixed model analysis of variance (ANOVA) was used to compare foot dimensions between various groups (including sex and age, as well as left and right foot). The participants were entered in the statistical model as a random effect. Gender, age, and side (left or right) were treated as fixed effects. For post hoc testing, Fisher least Significant (LSD) difference testing was used. Statistical significance was accepted when *p* < 0.01.

## Results

Foot measurements of six hundred and ninety-eight school children (*N* = 698) (431 girls; 267 boys) between the ages of six and sixteen years were taken. The average age of the participants was 10.86 (SD = 2.55). Table 2 shows the number and gender of each age group tested with age specific differences in foot sizes (HTL, FW, left and right foot) between boys and girls.

**Table 2:** Number of children tested with age and gender specific differences in foot sizes

Age (years)	N	Gender	Differences in HTL between G and B	Differences in FW between G and B	Differences in foot length between L and R feet	Differences in foot width between L and R feet
6	22	G:12; B:6			Right foot significantly longer than left foot in both genders and all ages (*p* < 0.01)	
7	94	G:51; B:37				Right foot significantly wider than left foot in both genders from age of 7 years (*p* < 0.01)
8	61	G:28; B:33				
9	46	G:27; B:17				
10	71	G:40; B:30				
11	78	G:43; B:34		Boys significantly wider feet than girls (ages 11 to 14) (*p* < 0.01)		
12	155	G:101; B:49	Boys significantly longer feet than girls (ages 12 to 16) (p < 0.01)			
13	93	G:56; B:31				
14	67	G:43; B:19				
15	36	G:26; B:7				
16	8	G:4; B:4				
6—16	698	G:431; B:267				

*N* Number of participants, *G* Girl participants, *B* Boy participants, *HTL* Heel-toe-length, *FW* Foot width

**Fig. 4:**
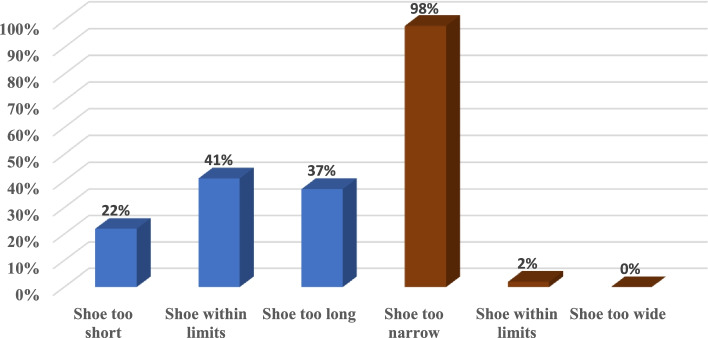
Percentage of children within each shoe-fit category based on shoe length and width

Foot measurements of HTL and FW (with an added toe allowance of 10 mm) were compared to the range of SL and SW measurements of new shoes in the corresponding shoe size. The percentage of children whose foot measurements were either too short, too long, too narrow, too wide or within limits compared to their corresponding school size are shown in Fig. 4. Values are shown as a percentage of the whole sample.

Twenty-two percent (22%) (*N* = 154) of the children tested wore shoes too short, forty-one percent (41%) (*N* = 286) wore shoes within limits and thirty-seven percent (37%) (*N* = 258) wore shoes that were too long. Concerning the SW, (with allowance of 10 mm), ninety-eight percent (98%) (*N* = 682) of the shoes were too narrow for the feet, two percent (2%) (*N* = 15) were within limits, and zero percent (0%) (*N* = 1) were too wide for the feet.

## Discussion

The current study aimed to determine if the dimensions of the currently available prescribed school shoes accommodate the dimensions (length and width) in a sample of habitually barefoot children and adolescents. The first important finding was that for ninety-eight percent (98%) of the participants, school shoes were too narrow for their feet. This finding supports previous research, which indicated that sixty-six-point seven percent (66.7%) of participants from Spain wore shoes that were too narrow [14]. The difference in results between the Spanish and the current South African study could be ascribed to the footwear habits (habitually barefoot) of South African children and adolescents [14]. South Africa is regarded as a country with a warm climate and children are barefoot more often than in other countries with colder weather, which trend possibly also influences the shape and development of the foot [18]. Researchers agree that habitually barefoot populations displayed an increased or above-average forefoot width, compared to habitually shod populations [7, 8, 23, 26, 28]. In the current study, SW was measured with a sliding caliper to measure the widest part on the outside of the shoe, while previous studies measured the inner SW with specialized telescopic gauges containing protractors [14]. Elena et al*.*[14] as well as the current study determined SW fit with an added 10 mm for width fit allowance. Measuring on the outside of the shoe has the limitation of the shoe material, adding to the width of the shoes. This would, however, mean that the added width of the shoe, due to the shoe material, especially supports the finding that the currently available shoes are not wide enough for the children’s feet.

In the current study, fifty-nine percent (59%) of children wore shoes that were not the correct length. Considering a recent study on shoe fit on 505 school going children in Spain, also taking into account a toe allowance of 5–15 mm, seventy-two-point five percent (72.5%) of school-going children were wearing ill-fitting shoes [14]. These findings correlate with a previous study conducted in South Africa which reported sixty-seven percent (67%) of South African children [26] wearing ill-fitting shoes. A possible reason for the differences between the current study and the previous studies is that previous studies measured the shoe after it had been worn by the participants. This poses the question whether, at the time the previous research was conducted, the shoes were in good condition or not, and if that factor could have contributed to the percentage of ill-fittings discovered. The current study investigated the shoe measurements using a newly manufactured shoe. It is, therefore, challenging to compare the results obtained from using a newly manufactured shoe to that of one that has been worn for a period. Some arguments could be made that the percentage of ill-fitting shoes in the previous studies might have been either lower or higher had the shoe been measured before it was worn for some time.

Upon further inspection of the school shoes currently available in the South African retail market, it was found that a good range of school shoe lengths are available. The reason for ill-fitting shoes, when considering HTL, might be the quality of information available on footwear fit, which can, at times, be scarce and not scientifically based [13]. Other reasons for ill-fitting shoes in HTL might be the rapid increase in HTL in children between the ages of six and fourteen years of age. Between these ages, HTL in children can increase up to 1.5 cm (centimetre) per year, after which it reaches a plateau [5, 20, 26]. Therefore, the shoe fit needs to be checked regularly while keeping the appropriate toe allowance in mind. Accurate feedback on the shoe fit, concerning toe allowance, cannot be obtained from the child, therefore, parents need to be educated appropriately to select the appropriate shoe size [15]. Even though a toe allowance of 10 mm has been added the to the HTL results, the current study still reports thirty-seven percent (37%) of shoes being too long for the foot. This correlates with the findings of Barisch-Fritz et al*.*[15] that toe allowance should be smaller than assumed. The recommended 90th percentile for toe allowance for females is 9.8 mm and for males 11.5 mm [15]. Another possible reason for shoes not corresponding with the HTL might be the decision to change the SL in an attempt to accommodate the width of the foot for more comfort, as South African footwear do not offer a width adjustment option.

It is debatable if the children merely chose the wrong shoes size due to limited information available on footwear fit. It needs to be pointed out that even if the correct school shoe size with regards to SL was selected, the SW in the ninety-eight percent (98%) of FW measurements gathered in this study would not have been catered for.

The current study supports the findings of Mauch et al*.*[18] that intercontinental differences in foot dimensions are common. The reasons behind the differences might require more research. It is essential for the shoe industry to familiarize itself with these research-based differences and adapt footwear accordingly to ensure better-fitting shoes. Upon investigation, it appears that shoe companies are not currently catering for inter-continental feet differences. Previous studies, which investigated foot shapes and morphological differences between habitually barefoot children and habitually shod children, reported longer feet in the barefoot population [7]. This finding is supported by Shu et al*.* [35], who reported significantly larger feet in barefoot females. These results, however, do not align with the finding of a study on German habitually shod children, which reported significantly longer feet than their Australian, habitually barefoot, counterparts. This difference was, however, only present in younger children, and no significant difference was recorded for older subjects [18].

While the adult foot has been researched extensively, the pediatric foot is still a much-understudied field [29]. Even when looking at the external foot shape of a child, when compared to an adult, the structural and functional characteristics are different [22]. Anthropometrical data on the pediatric foot is important when considering footwear design and shoe construction [21, 29, 36, 37]. The maximal forces and leverages of children’s feet are vastly different from those of adults. Subsequently, a shoe which provides cushioning and stability to most adults, may feel hard and uncomfortable for children [21].

The market for children’s shoes is not currently driven by orthopaedic and biomechanical considerations, but by consumer behaviour and trends. Children’s shoes are mostly developed as downsizing of an adult shoe [21]. According to previous studies, manufacturers are not taking the 3D shape of the foot into account and are unable (or unwilling) to produce a variety to cater for the differences in foot morphology [22, 29].

Shoe designs for children in habitually barefoot populations should, therefore, produce a shoe to fit the foot properly and mimic the shape and dimensions of the bare foot. Optimum foot development occurs when the natural shape and function of the foot is respected [8, 21, 29, 36, 37]. However, one of the most challenging tasks that remain in shoe manufacturing is the ability to access useful data to build a standard shape from available measurements [30]. In most current designs, SL is used as a basic measurement, without considering the other dimensions [30].

Ill-fitting shoes can have a lasting effect on the gait patterns and development of the foot, causing foot abnormalities such as a hammer-, clawed- and retracted toes [30, 39]. Associated pain and pathology, due to ill-fitting footwear, are widely reported [11, 13, 16, 28]. Previously, eighty percent (80%) of South African adult females reported wearing shoes, which caused pain, blisters and callouses [28]. Common foot deformities like hallux valgus are significantly induced and influenced by shoe fit being too narrow or too short [30, 39]. Shoes, which are too narrow for the foot, will restrict the slay of the forefoot, leading to biomechanical deviations and restriction in movement of the foot [10]. Hallux valgus are believed to be avoided and corrected by selecting and wearing shoes that provide sufficient space for the toes [30].

A limitation of the current study could be the assessment of only two static measurements, being the HTL and FW compared to the SL and SW and not including a foot height measurement. However, school shoes in the South African retail market have the required shoelaces and/or adjustable straps over the upper part of the foot. The size over the upper part of the foot can therefore be adjusted. Using a pedobarographic platform to ensure the body weight was distributed exactly 50% over each foot while measuring in the standing position could have added value to the data collection procedures. Pedobarographic data would ensure that the foot is in maximal elongated position during the standing measurement.

Dynamic measurements will add another dimension to the development of the South African pediatric shoe. Future research to investigate the foot dimensions of habitually barefoot children and adolescents, focusing on shoe development, under dynamic conditions, is indicated. Future research should also investigate whether currently available shoes respect the foot development of the habitually barefoot population, with a specific focus on foot pliability and arch height index.

## Conclusions

The current study advances and elaborate on existing views on this highly relevant issue, as it places the onus on shoe manufacturing companies to enhance and improve on the basic dimensions of available shoes for habitually barefoot populations. Given the width dimensions of shoes available in the present study, the majority of children in South Africa will not have the option of a school shoe with a wider forefoot, as this is not available.

Results from the current study should create awareness of the current mismatch between the children’s feet and available school shoes. Results could assist the shoe manufacturing industry, to provide well-fitting shoes for habitually barefoot populations. For the immediate future, better fitting school shoes can assist with healthy foot development in children. In the long term, access to well-designed and foot-appropriate shoes could lessen foot deformities, pain and musculoskeletal injuries in adulthood.

## Data Availability

The datasets used and/or analysed during the current study are not publicly available due to it currently being used in negotiations with local shoe manufacturing companies but are available from the corresponding author on reasonable request.

## Abbreviations

%: Percentage
SD: Standard deviation
BMI: Body mass index
cm: Centimetre
FW: Foot width
HTL: Heel-toe-length
ICC: Interclass correlation coefficient
LSD: Fisher least Significant
mm: Millimetre
MTH1: First metatarsal head
MTH5: Fifth metatarsal head
N: Number
R: South African Rand
SL: Shoe length
SW: Shoe width
UK: United Kingdom
